# Flame-Retardant and Hydrophobic Cotton via Alkoxysilyl-Functionalized Polysiloxanes, Cyclosiloxanes, and POSS with Surface Thiol-Ene Dithiophosphate Grafting

**DOI:** 10.3390/ma19020265

**Published:** 2026-01-08

**Authors:** Marcin Przybylak, Anna Szymańska, Weronika Gieparda, Mariusz Szołyga, Agnieszka Dutkiewicz, Hieronim Maciejewski

**Affiliations:** 1 Poznań Science and Technology Park, Adam Mickiewicz University Foundation, Rubież 46, 61-612 Poznań, Poland; anna.szymanska@ppnt.poznan.pl (A.S.); mariusz.szolyga@ppnt.poznan.pl (M.S.); agnieszka.dutkiewicz@ppnt.poznan.pl (A.D.); 2 Department of Bioproduct Engineering, Institute of Natural Fibers & Medicinal Plants, National Research Institute, Wojska Polskiego 71b, 60-630 Poznań, Poland; weronika.gieparda@iwnirz.pl; 3 Faculty of Chemistry, Adam Mickiewicz University, Uniwersytetu Poznańskiego 8, 61-614 Poznań, Poland

**Keywords:** flame retardant, cotton, organosilicon, thiol-ene, hydrophobic

## Abstract

In this work, a multifunctional surface engineering strategy was developed to impart both flame-retardant and hydrophobic properties to cotton fabrics. In the first stage, cellulose fibers were modified with poly(methylvinyl)siloxane containing trimethoxysilyl groups, 2,4,6,8-tetramethyl-divinyl-bis(trimethoxysilylpropyltioethyl)cyclotetrasiloxane, or tetrakis(vinyldimethylsiloxy)tetrakis(trimethoxysilylpropyltioethyl)octasilsesquioxane (POSS). All modifiers contained alkoxysilyl groups capable of forming covalent bonds with cellulose hydroxyl groups. The modification was performed using a dip-coating process followed by thermal curing. This procedure enabled the formation of Si-O-C linkages and the generation of a reactive organosilicon layer on the cotton surface. In the second step, O,O′-diethyl dithiophosphate was grafted directly onto the vinyl-functionalized fabrics via a thiol-ene click reaction. This process resulted in the formation of a phosphorus- and sulfur-containing protective layer anchored within the siloxane-based network. The obtained hybrid coatings were characterized using Fourier-transform infrared spectroscopy (FT-IR), scanning electron microscopy (SEM), and SEM-EDS. These analyses confirmed the presence and uniform distribution of the modifiers on the fiber surface. Microscale combustion calorimetry demonstrated a substantial reduction in the heat release rate. Thermogravimetric analysis (TG/DTG) revealed increased char formation and altered thermal degradation pathways. The limiting oxygen index (LOI) increased for all modified fabrics, confirming enhanced flame resistance. Water contact angle measurements showed values above 130°, indicating effective hydrophobicity. As a result, multifunctional textile surfaces were obtained. In addition, the modified fabrics exhibited partial durability toward laundering and retained measurable flame-retardant and hydrophobic performance after repeated washing cycles.

## 1. Introduction

Natural cotton fabrics are widely used due to their comfort, breathability, softness, biodegradability, and origin from renewable resources, which makes them attractive for sustainable textile applications. At the same time, the hydrophilic character arising from the abundance of hydroxyl groups in cellulose promotes water absorption, soiling, microbial growth, dimensional changes, and deterioration of mechanical performance, which limits their use in technical, outdoor, and protective textiles unless the surface is chemically modified [1,2]. Cotton fabrics are inherently highly flammable: pure cotton exhibits a low limiting oxygen index, ignites easily, burns rapidly without self-extinguishing, and produces only minimal char during thermal degradation, which poses a significant safety risk in clothing and interior textile applications [3,4]. Therefore, there is a strong need to develop finishing systems that simultaneously improve water and dirt repellency, and reduce flammability while maintaining handle, breathability, and wash durability [5].

Traditional flame-retardant strategies for cellulose-based textiles relied heavily on halogenated salts and compounds, including brominated ones. These systems operated primarily in the gas phase by releasing hydrogen halides that interfere with radical chain reactions during combustion. However, due to environmental persistence, bioaccumulation, and high smoke/toxicity generation, regulations like the Stockholm Convention have largely phased them out from textile applications [6,7]. Inorganic phosphorus-based flame retardants remain widely used, encompassing ammonium phosphates such as diammonium hydrogen phosphate (DAHP), ammonium dihydrogen phosphate (ADHP), and monoammonium phosphate (MAP). These catalyze cellulose dehydration in the condensed phase, promoting char formation, while reducing volatile flammable gases. Red phosphorus and phosphorus oxides also contribute by forming protective polyphosphoric acid layers during thermal decomposition [8,9,10]. Organic phosphorus compounds dominate modern halogen-free systems, including phosphonates (e.g., dimethyl methylphosphonate), phosphates (e.g., triethyl phosphate), and polyphosphates like ammonium polyphosphate (APP). Reactive derivatives such as DOPO (9,10-dihydro-9-oxa-10-phosphaphenanthrene-10-oxide) and its analogs (e.g., DOPO-phenol, DOPO-urea) provide covalent bonding to cellulose, acting in both gas (radical scavenging) and condensed phases (char promotion). Phosphoramidates and phosphonic acids further enhance intumescence through acid-catalyzed depolymerization [11,12,13,14]

Organosilicon compounds such as alkoxysilanes, polysiloxanes, and polyhedral oligomeric silsesquioxanes (POSS) are widely used to modify cotton because they combine a thermally stable Si-O-Si backbone with tunable organic substituents, low surface energy, and good film-forming ability [15,16]. Alkoxysilyl groups hydrolyze and condense to form siloxane networks that are covalently anchored to cellulose, providing durable finishes that can be engineered for hydrophobicity, flame retardancy, antimicrobial activity, or combinations thereof [17,18]. Polysiloxanes bearing trialkoxysilyl groups have been shown to form flexible, wash-resistant coatings that significantly increase water contact angle, and when combined with phosphorus-containing can simultaneously improve flame retardancy and impart additional functions. [19,20,21] Cage-like silsesquioxanes (POSS) with alkoxysilyl substituents and long hydrocarbon or fluorinated chains have been successfully applied to cotton, where their nano-structured cores and tailored substituent patterns allow control of surface roughness, surface energy, and char structure, enabling superhydrophobicity and, in some cases, enhanced thermal stability and flame resistance [22,23,24]. Recent studies on polysiloxanes and silsesquioxanes containing P, S, and Si show that integrating phosphorus and sulfur into organosilicon frameworks provides strong synergistic effects in condensed phase and gas phase, leading to large HRR reductions and high char yields at relatively low add-on levels [25,26].

Thiol-ene click chemistry has emerged as a versatile and efficient tool for preparing organosilicon derivatives and for direct functionalization of cotton surfaces. Radical hydrothiolation of vinyl-substituted silanes, polysiloxanes, or POSS proceeds under mild conditions, often at room temperature and without metal catalysts, and tolerates a wide range of functional thiols including fluorinated chains, long alkyl groups, alkoxysilyl fragments, and phosphorus-containing moieties [27,28]. On textile substrates, thiol-ene reactions are typically applied either after introducing SH groups with 3-mercaptopropyltrialkoxysilanes or by using vinyl-functionalized organosilicon modifiers, enabling UV-induced grafting of hydrophobic chains or flame-retardant fragments directly on the fiber surface with high efficiency and excellent wash durability [29,30]. Several reports have demonstrated that combining thiol-ene functionalization with POSS or polysiloxane architectures allows the construction of superhydrophobic, mechanically robust, and sometimes flame-retardant cotton fabrics, while the incorporation of phosphorus- and sulfur-bearing thiols yields P/S/Si systems with strong synergistic effects on char formation and HRR reduction. Importantly, previous work has also shown that the way thiol-ene reactions are carried out (bulk vs. on-fabric) and the steric environment around vinyl groups critically influence the orientation of hydrophobic chains, the degree of crosslinking, and ultimately the surface and flame-retardant properties of the fabrics [31,32,33].

The existing literature on cotton modification with silanes, polysiloxanes, silsesquioxanes (POSS), and P/S/Si systems clearly demonstrates that individual approaches can deliver durable hydrophobicity or significant flame retardancy, but achieving both effects simultaneously at low add-on, without compromising fabric handle, remains challenging. Previous studies using thiol-ene chemistry have mainly focused either on hydrophobization with organosilicon derivatives or on introducing phosphorus-based flame retardants, and the influence of the organosilicon scaffold (linear polysiloxane vs. cyclosiloxane vs POSS) on the balance between hydrophobic and flame-retardant performance has not been systematically compared under identical conditions. In particular, there is a lack of data on difunctional systems where alkoxysilyl-bearing vinyl polysiloxanes, cyclic siloxanes, or POSS are combined with O,O′-diethyl dithiophosphate via thiol-ene reaction directly on the cotton surface, creating P/S/Si-rich networks with controlled numbers and distributions of anchoring points. Therefore, the present work addresses this gap by designing and applying trimethoxysilyl-functionalized poly(methylvinyl)siloxane, a vinyl cyclosiloxane and a vinyl POSS, each partially substituted with alkoxysilyl groups and further functionalized with O,O′-diethyl dithiophosphate through thiol-ene click chemistry on cotton fabrics, and by evaluating how the architecture of the organosilicon modifier governs the resulting hydrophobicity and flame-retardant performance at comparable add-on.

## 2. Materials and Methods

### 2.1. Materials

All organosilicon modifiers used in this study were synthesized in-house according to procedures adapted from our previous research and relevant literature [34,35,36]. Acetic acid (99.5%) and tetrahydrofuran (99%) were purchased from Sigma Aldrich (Poznan, Poland), while dodecanethiol (98%) was supplied by Acros Organics (Gell, Belgium). A bleached cotton fabric produced by the Textile Factory in Łódź (Poland) served as the substrate. The material was woven in a plain weave and had an areal density of 150 g/m^2^.

### 2.2. Analytical Methods

#### 2.2.1. FT-IR Spectroscopy

The chemical structure of modified cotton was examined using a Bruker Tensor 27 FT-IR spectrometer (Rheinstetten, Germany) equipped with a Specac Golden Gate single-reflection diamond ATR accessory. All spectra were acquired at room temperature.

#### 2.2.2. Contact Angle Measurements

Static water contact angles (WCA) and oil contact angles (OCA) were measured using a Krüss DSA100 Expert goniometer (Hamburg, Germany). The system featured an automated XYZ stage, four-channel dosing unit, and a high-resolution camera with adjustable optical settings. Droplets (5 µL) of deionized water were used for WCA, whereas droplets of hexadecane, motor oil, mineral oil, and pump oil were applied for OCA measurements. Each value represents the mean of five independent measurements taken at different positions on the fabric.

#### 2.2.3. SEM-EDS Analysis

Surface morphology and elemental composition were assessed using a Hitachi SU-3500 scanning electron microscope equipped with an energy-dispersive X-ray detector (Tokyo, Japan). Samples were sputter-coated with gold prior to analysis.

#### 2.2.4. Microscale Combustion Calorimetry (MCC)

Flammability characteristics were determined using a Fire Testing Technology (FTT) pyrolysis-combustion flow calorimeter. Samples (5–6 mg) were heated at 1 °C s^−1^ from 75 to 750 °C under nitrogen/oxygen (80/20 cm^3^ min^−1^), while combustion occurred at 900 °C. Three replicates were tested; HRR uncertainty was ±2%.

#### 2.2.5. Limiting Oxygen Index (LOI)

LOI values were measured according to the standard [37] Each specimen was tested three times; the measurement error was ±0.5%.

#### 2.2.6. Thermogravimetric Analysis (TGA)

Thermal decomposition was examined using a TA Instruments Q50 apparatus (TA Instruments—a division of Waters Corporation, New Castle, DE, USA). Samples (9–10 mg) were heated in synthetic air (60 mL min^−1^) from room temperature to 700 °C at 10 °C min^−1^. The instrumental errors were 0.5% for sample mass and ±1 °C for temperature.

### 2.3. Synthesis of Organosilicon Compound

To obtain organosilicon modifiers with a defined number of hydrolysable alkoxysilyl groups, three representative compounds, a polysiloxane, a POSS derivative, and a cyclic tetramer were synthesized using UV-initiated thiol-ene chemistry. In each case, (3-mercaptopropyl)trimethoxysilane served as the thiol component, ensuring the introduction of alkoxysilyl functionalities capable of forming covalent Si-O-C linkages with cellulose. The polysiloxane was obtained by reacting poly(methylvinyl)siloxane with (3-mercaptopropyl)trimethoxysilane via a UV-initiated thiol-ene addition, carried out in the presence of 2,2-dimethoxy-2-phenylacetophenone (DMPA) as the photoinitiator [34]. The POSS derivative was synthesized through a thiol-ene reaction between octavinylsilsesquioxane (POSS, (ViSiO_1_._5_)_8_) and (3-mercaptopropyl)trimethoxysilane under UV irradiation, using (DMPA) to initiate the addition [35].

The cyclic siloxane modifier was prepared by reacting 2,4,6,8-tetramethyl-2,4,6,8-tetravinylcyclotetrasiloxane (D_4_-Vi) with (3-mercaptopropyl)trimethoxysilane via UV-promoted thiol-ene addition in the presence of (DMPA), enabling selective incorporation of alkoxysilyl groups [36].

### 2.4. Modification of Cotton Fabrics

The modification of cotton fabrics was carried out in two sequential stages. In the first step, the synthesized alkoxysilyl-functionalized organosilicon products, namely the polysiloxane derivative, the functionalized silsesquioxane (POSS), and the functionalized cyclic siloxane derived from (D_4_), were applied to the fabrics from a hydrolyzed compounds bath. Each compound was subjected to hydrolysis condensation in a solution containing 5 wt.% silane, 5 wt.% acetic acid, 5 wt.% water, and 85 wt.% tetrahydrofuran (THF). The mixture was stirred for 1 h at ambient temperature, after which cotton samples were immersed for 30 min. Excess liquid was removed with absorbent paper, followed by drying at 80 °C for 1 h and thermal curing at 130 °C for 3 min.

In the second step, the silane-modified fabrics were functionalized with O,O′-diethyl dithiophosphate via a thiol-ene photochemical reaction. A 10 wt.% solution of O,O′-diethyl dithiophosphate was prepared and supplemented with 2,2-dimethoxy-2-phenylacetophenone (DMPA) as the photoinitiator. The solution was applied onto the previously modified fabrics by spray coating to ensure uniform distribution of the reagent; to guarantee reproducibility, 4 mL of the solution was sprayed onto each fabric sample of 8 × 11 cm, and the applied volume was adjusted when necessary, so that the amount of modifier per unit area (cm^2^) remained constant. The samples were then exposed to UV irradiation using an OmniCure S2000 lamp (wavelength range 250–600 nm) positioned at a fixed distance of 20 cm from the fabric surface, with an exposure time of 1 h to complete the surface thiol-ene grafting process. After illumination, the fabrics were rinsed with isopropanol to remove unreacted species and dried at room temperature.

### 2.5. Washing Process

To evaluate coating durability, the modified samples were subjected to repeated laundering according to PN-EN ISO 105-C06:2010, using detergent at 40 °C for 60 min, followed by intensive rinsing. The entire washing procedure was repeated ten consecutive times to assess the stability of the applied finishing.

### 2.6. Determination of Add-On

The add-on value was calculated using:

(1) $$Ad\mathrm{d-on}=\;\frac{m_{1}-\;m_{0}}{\;m_{0}}\times \;100$$

where add-on refers to the add-on value [%], m_0_ is the weight of the cotton fabric before modification [g], and m_1_ is the weight of the cotton fabric after modification [g].

## 3. Results

**Figure 1:**
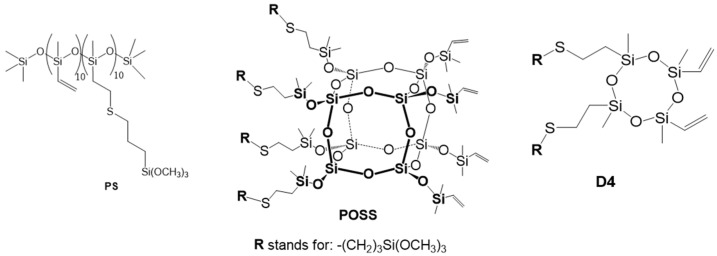
Chemical formula of organosilicon compounds.

Three organosilicon compounds were synthesized and applied as surface modifiers for cotton fabrics: poly(methylvinyl)siloxane-based polysiloxane (PS), octavinylsilsesquioxane (POSS), and 2,4,6,8-tetramethyl-2,4,6,8-tetravinylcyclotetrasiloxane (D_4_). Each compound was functionalized with alkoxysilyl substituents, which can undergo condensation with the hydroxyl groups of cellulose, enabling the formation of covalent Si-O-C bonds upon curing. All three modifiers were synthesized via thiol-ene click reactions, and in every case, approximately half of the original vinyl groups were substituted with alkoxysilyl functionalities. The chemical structures of the obtained organosilicon compounds are presented in Figure 1. The distribution of functional groups is statistical, and the formulas presented in the figure are for illustrative purposes only.

**Figure 2:**
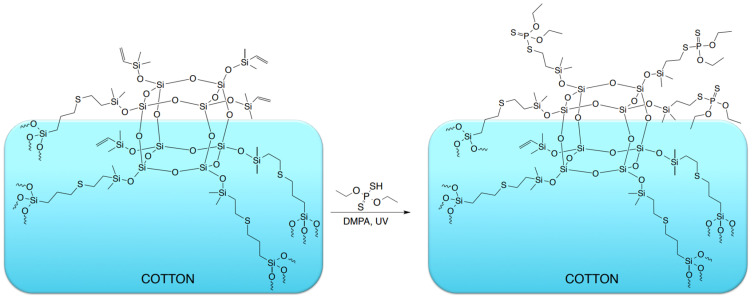
Schematic representation of the thiol-ene modification of cotton fabrics with POSS (sample C-POSS-P).

**Figure 3:**
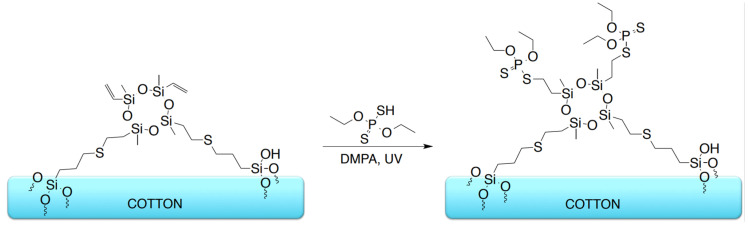
Schematic representation of the thiol-ene modification of cotton fabrics with D4 (sample C-D4-P).

**Figure 4:**
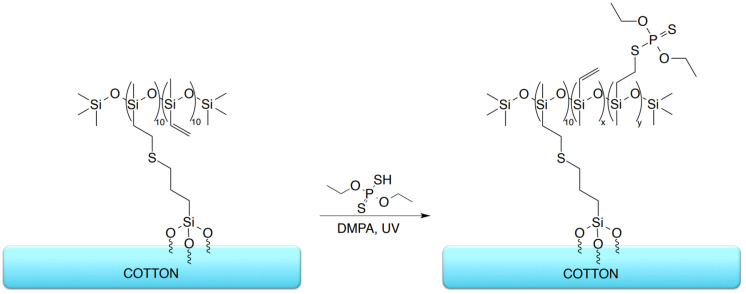
Schematic representation of the thiol-ene modification of cotton fabrics with polysiloxane (sample C-PS-P).

The organosilicon compounds were applied to cotton fabric using a dip-coating procedure, forming a reactive siloxane-based layer on the fiber surface. In the subsequent step, the modified fabrics were subjected to a thiol-ene reaction with O,O′-diethyl dithiophosphate, performed directly on the textile surface under UV irradiation, and the modification schemes for the organosilicon compounds as well as for the phosphorus-containing treatment are presented in Figure 2, Figure 3 and Figure 4.

Not all vinyl groups present in the organosilicon modifiers deposited on the fabric surface are expected to participate in the thiol-ene reaction, as steric constraints within the surface-bound siloxane layer can restrict the accessibility of some unsaturated sites. Accordingly, the reaction schemes presented in Figure 2 and Figure 3 deliberately depict residual vinyl groups, illustrating the partial conversion anticipated under the applied surface-grafting conditions and consistent with the experimental observations.

The designations of all prepared samples, together with their corresponding add-on values measured before and after laundering, are summarized in Table 1.

The add-on values obtained for all samples before and after laundering are presented in Table 1. Among the fabrics modified exclusively with organosilicon compounds, the highest mass increase was observed for the sample treated with silsesquioxane (C-POSS, 9.2%), followed by polysiloxane (C-PS, 8.8%) and cyclotetrasiloxane (C-D4, 8.7%). The slightly higher add-on value observed for the silsesquioxane (POSS) derivative may result from the presence of additional silicon atoms in the functional groups attached at the corners of the Si_8_O_12_ cage, which increases the overall inorganic fraction of the molecule compared with the linear polysiloxane (PS) and the cyclic siloxane (D_4_). Despite this structural difference, all three modifiers were designed so that exactly half of their vinyl groups were substituted with alkoxysilyl functionalities. This ensured that each compound possessed comparable numbers of hydrolysable Si(OCH_3_)_3_ groups, providing an equivalent opportunity for condensation with the cellulose surface. Therefore, the overall ability of each compound to bind to the fabric was intentionally kept similar. For this reason, the add-on values obtained for PS, POSS, and D_4_ differ only slightly and are not correlated with the molecular weight of the compounds, but instead reflect the controlled degree of functionalization.

Laundering did not reduce the add-on values of fabrics modified solely with the organosilicon compounds (C-PS, C-POSS, and C-D4). These results indicate that the siloxane-derived layers remained chemically bonded to cellulose and were not removed during washing. This behavior aligns with our previous findings, where alkoxysilyl-functionalized polysiloxanes and cyclic siloxanes showed excellent washing durability due to efficient formation of Si-O-C linkages and condensation-driven siloxane network stabilization at the fiber surface. In contrast, samples additionally functionalized with O,O′-diethyl dithiophosphate exhibited a moderate decrease in add-on after laundering. The reduction ranged from approximately 1.3–1.6 percentage points, consistent across all three organosilicon matrices (C-PS-P, C-POSS-P, C-D4-P). This decrease is attributed to the partial removal of the fraction of the O,O′-diethyl dithiophosphate that remained only physically deposited on the fiber surface and did not participate in the thiol-ene reaction. Similar observations have been described in flame-retardant systems where phosphorus-based additives demonstrate partial water solubility or limited interfacial anchoring, leading to gradual loss under repeated laundering [38].

**Figure 5:**
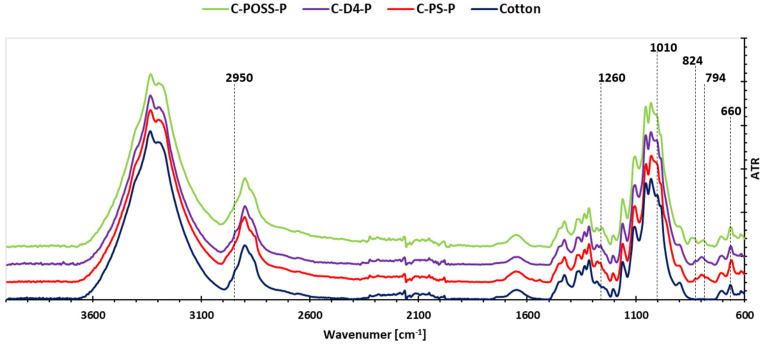
FT-IR spectra of modified samples before washing.

**Figure 6:**
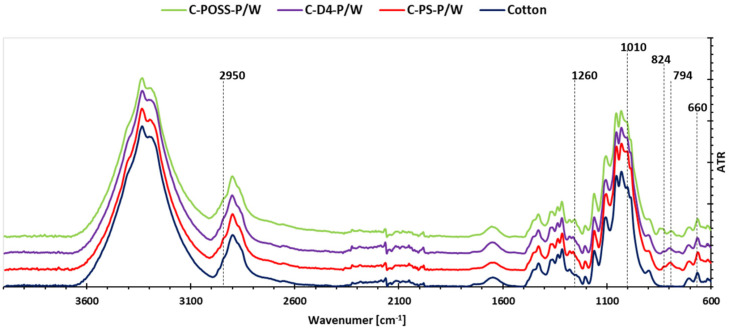
FT-IR spectra of modified samples after washing.

To verify the presence of the applied modifiers on the cotton surface, Fourier-transform infrared (FT-IR) spectroscopy was performed for both unwashed and washed samples. This analysis made it possible to assess not only the successful deposition of the organosilicon compounds and the phosphorus-containing layer, but also the durability of the formed coatings after repeated laundering. The corresponding FT-IR spectra for the untreated and laundered fabrics are presented in Figure 5 and Figure 6.

The FT-IR spectra recorded for the modified cotton fabrics before washing (Figure 5) and after washing (Figure 6) confirm the successful deposition of the organosilicon compounds and the subsequent grafting of O,O′-diethyl dithiophosphate onto the fiber surface. All modified samples display additional absorption bands that are absent in the spectrum of unmodified cotton, demonstrating the presence of polysiloxane-, POSS-, or D_4_-derived structures as well as phosphorus-containing functionalities. Characteristically, all spectra exhibit a band at 2950 cm^−1^, attributed to the CH stretching vibration of CH_3_. A distinct band associated with Si-CH_3_ groups appears near 1260 cm^−1^. The region 950–1100 cm^−1^ shows a broad composite signal arising from the overlapping contributions of Si-O-Si, POC_2_H_5_, and the intrinsic cellulose backbone. The most intense band within this region, observed around 1010 cm^−1^, corresponds to POC_2_H_5_. Bands characteristic of organophosphorus functionalities are visible at 824 cm^−1^, 795 cm^−1^, and 660 cm^−1^, corresponding to P=S and P-S vibrations. However, the bands at 824 cm^−1^ and 795 cm^−1^ partially overlap with the typical cellulose-derived Si-O-C vibration that appears at 794 cm^−1^, as noted in previous studies on silane-modified cotton fabrics [34].

After laundering, the FT-IR spectra of the washed samples (C-PS-P/W, C-POSS-P/W, C-D4-P/W) maintain the same characteristic bands observed for their unwashed counterparts, indicating the persistence of both organosilicon and phosphorus-containing components on the fabric. A slight reduction in the intensity of selected peaks, particularly those associated with the phosphorus groups, can be observed, which is consistent with the partial removal of O,O′-diethyl dithiophosphate during washing. Nevertheless, the overall spectral profiles remain comparable, confirming that the primary siloxane-based coatings exhibit high resistance to laundering and retain their chemical integrity.

To further confirm the presence and distribution of the deposited modifiers on the cotton surface, scanning electron microscopy coupled with energy-dispersive X-ray spectroscopy (SEM-EDS) was performed for all modified samples. This analysis enabled visualization of morphological changes induced by the organosilicon coatings, as well as elemental verification of silicon-, phosphorus-, and sulfur-containing species introduced during the two-step modification process. The results of the EDS measurements are summarized in Table 2.

The EDS analysis confirms the successful deposition of the organosilicon modifiers and the phosphorus-containing layer on the cotton surface. All treated samples exhibit the characteristic elements originating from the applied compounds, most notably silicon in fabrics modified with polysiloxane, POSS, and D_4_. Sulfur is also present in all modified samples, as it originates from the thiol-ene reactions used to synthesize each organosilicon modifier; however, its content increases further after grafting with O,O′-diethyl dithiophosphate, which introduces additional S atoms through its P=S and P-S groups. Phosphorus (P) appears exclusively in the samples subjected to the second modification step, confirming the successful attachment of the phosphorus-containing layer. Unmodified cotton contains only carbon and oxygen and therefore serves as a clean reference. For fabrics modified solely with the organosilicon compounds (C-PS, C-D4, and C-POSS), the elemental composition remains essentially unchanged after washing. This confirms the high stability of the siloxane-based coatings and their strong covalent attachment to the cellulose surface. Among these three systems, the POSS-modified fabric contains slightly more silicon, which is consistent with the molecular structure of silsesquioxane (POSS). The samples subjected to the two-step modification involving thiol-ene grafting of O,O′-diethyl dithiophosphate (C-PS-P, C-D4-P, and C-POSS-P) contain clearly detectable phosphorus and sulfur, verifying the presence of the phosphorus-containing layer. Importantly, the S signal detected by EDS arises from two distinct sources: (i) the covalently bound (3-mercaptopropyl)trimethoxysilane segments that are incorporated into the organosilicon modifiers during the initial thiol-ene synthesis step, and (ii) the additional dithiophosphate groups introduced in the second thiol-ene grafting stage, so that the increase in sulfur observed for C-PS-P, C-D4-P, and C-POSS-P relative to C-PS, C-D4, and C-POSS can be attributed specifically to the presence of O,O′-diethyl dithiophosphate on the fiber surface. After washing, the amount of silicon remains nearly constant for all samples, confirming the robust and durable attachment of the organosilicon component. In contrast, the phosphorus and sulfur contents show a slight decrease for the P-modified fabrics, indicating partial removal of the O,O′-diethyl dithiophosphate layer during laundering. However, the persistent presence of both elements even after washing demonstrates that a significant portion of the phosphorus-containing modifier remains bound to the fabric.

**Figure 7:**
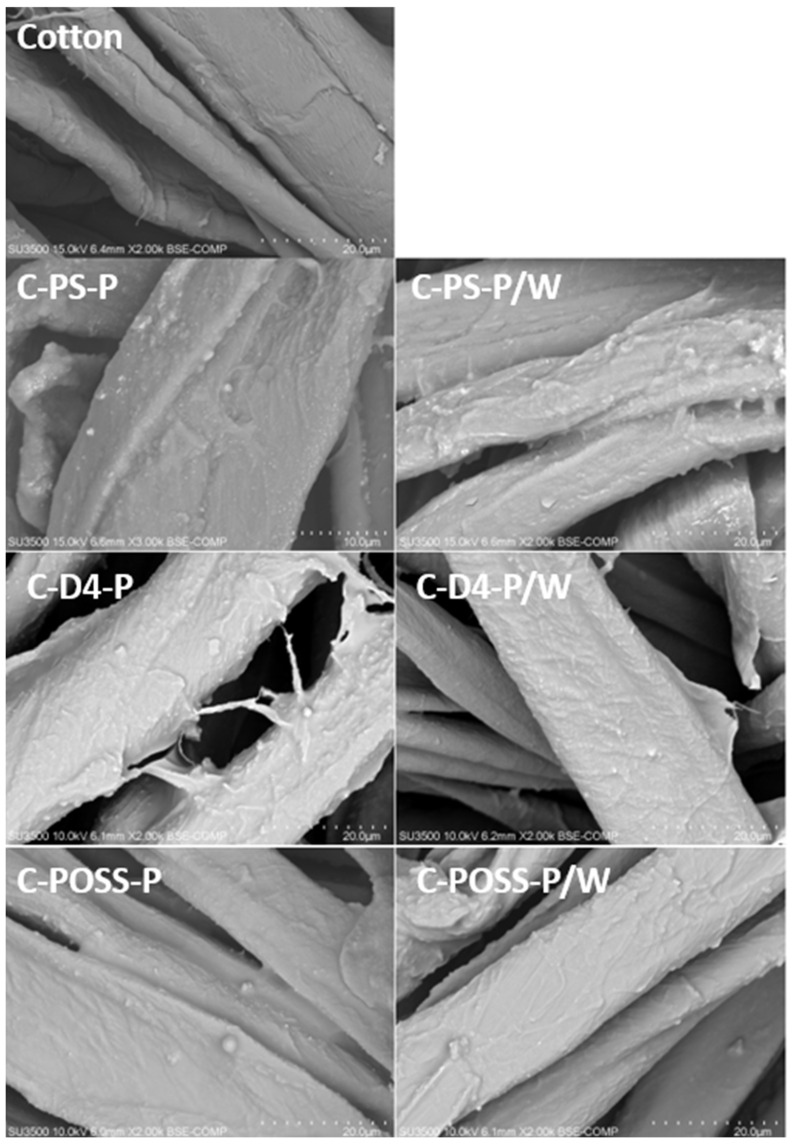
SEM image of modified samples before and after washing (W).

The next figure (Figure 7) presents SEM images of the modified cotton samples before and after washing, allowing direct visualization of the morphological effects of the applied treatments and the durability of the coatings under laundering conditions.

The SEM image of the unmodified cotton fabric shows the characteristic morphology of native cellulose fibers, including their longitudinal grooves and irregular surface typical of cotton. In contrast, all modified samples display a continuous and uniform siloxane-based coating covering the fiber surface. The deposited layer adheres tightly to the textile, shows no visible cracks or delamination, and forms a smooth, homogeneous film around individual fibers. Importantly, the SEM images of the washed samples reveal no noticeable changes in fiber morphology or coating integrity, indicating that the applied modification remains stable and well-preserved even after laundering.

**Figure 8:**
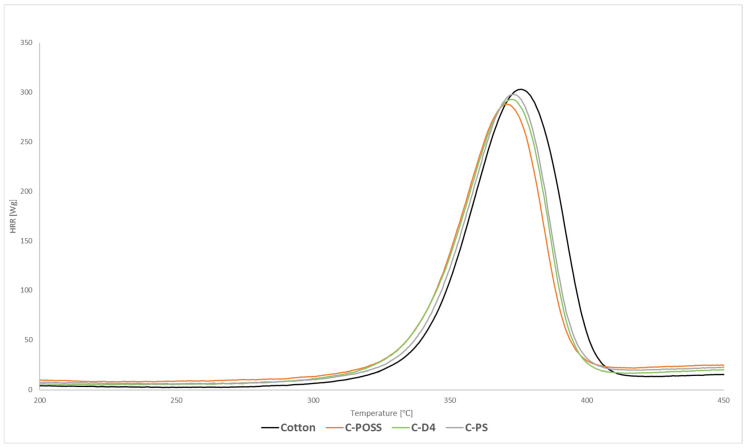
HRR curves of modified samples before grafting phosphate onto the cotton surface.

**Figure 9:**
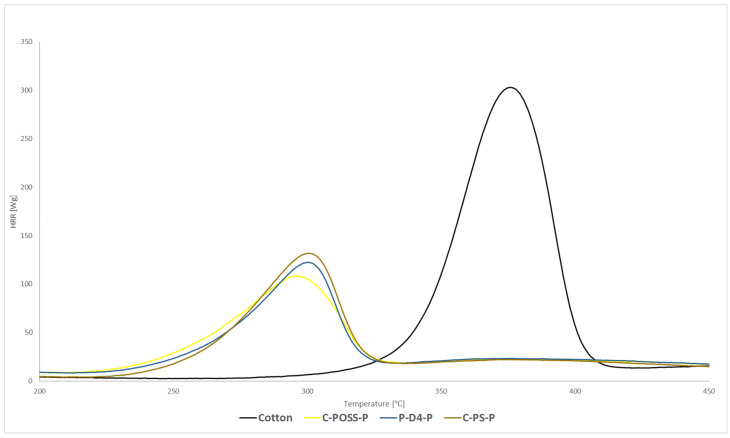
HRR curves of modified samples after grafting phosphate onto the cotton surface.

**Figure 10:**
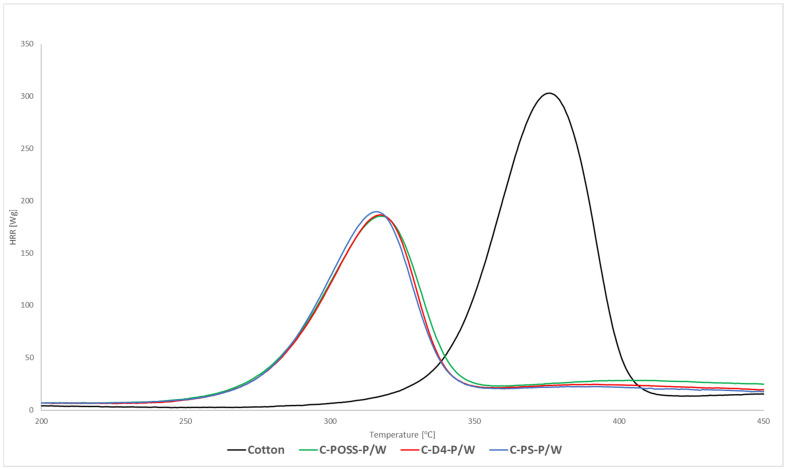
HRR curves of modified samples after grafting phosphate onto the cotton surface and after the washing process.

Flammability was first evaluated using microscale combustion calorimetry (MCC), and the corresponding heat release rate (HRR) curves for both unwashed and washed samples are presented in Figure 8, Figure 9 and Figure 10.

Microscale combustion calorimetry (MCC) revealed that modification of cotton with the organosilicon compounds alone resulted in only a modest reduction in the peak heat release rate (pHRR) compared with the untreated fabric. This limited improvement can be attributed to the substantial organic fraction present in polysiloxane, silsesquioxane (POSS), and cyclotetrasiloxane (D_4_), which contributes combustible species during thermal decomposition and thus partially counterbalances the protective role of the Si-O-Si domains formed upon curing. As summarized in Table 3, the pHRR values for C-PS, C-D4, and C-POSS remain close to that of raw cotton, confirming the limited standalone effect of the organosilicon layer. By contrast, samples subjected to the second modification step, in which O,O′-diethyl dithiophosphate was grafted via a thiol-ene reaction, exhibited a substantial decrease in pHRR, confirming the strong flame-retardant activity of the phosphorus- and sulfur-containing functionality. According to Table 3, pHRR values decreased from 304 W/g for raw cotton to 132, 122, and 108 W/g for C-PS-P, C-D4-P, and C-POSS-P, respectively. The most pronounced reduction was observed for the POSS-based sample C-POSS-P, whose pHRR decreased to 108 W/g-over 66% lower than that of the raw cotton, indicating highly effective condensed- and gas-phase inhibition (Table 3). Additionally, all modified samples showed a shift in the HRR maximum toward lower temperatures. As shown in Table 3, Tmax values decreased from 376 °C for cotton to approximately 296–300 °C for phosphorus-modified fabrics. This shift is considered beneficial because the earlier onset of decomposition promotes char formation at reduced temperatures, resulting in a smaller release of flammable volatiles and facilitating the development of a coherent protective residue. Such behavior is characteristic of systems in which condensed-phase stabilization dominates the flammability mechanism.

The hierarchy of flame-retardant performance among the organosilicon precursors—POSS being the most effective, followed by D_4_ and then polysiloxane—can be explained by their structural differences. POSS contains a rigid Si_8_O_12_ cage, which provides a high proportion of inorganic silicon and forms dense, silica-rich char layers that act as efficient thermal shields. Its globular architecture also favors uniform surface distribution and greater accessibility of unreacted vinyl groups, enhancing the efficiency of the subsequent thiol-ene grafting of the phosphorus-containing additive. The D_4_ derivative offers an intermediate level of rigidity: its cyclic structure promotes the formation of vitrified Si-O-Si networks during pyrolysis, resulting in better flame retardancy than linear polysiloxane but less than the highly inorganic POSS scaffold. Polysiloxane, being the most flexible and organic-rich structure, forms the least cohesive protective layer and provides fewer sterically accessible sites for thiol-ene modification, leading to the lowest improvement among the three. After laundering, washed samples displayed slightly higher pHRR values than their unwashed counterparts. As reported in Table 3, pHRR increased to 185–189 W/g after washing, reflecting partial loss of the phosphorus-containing component. Nevertheless, all washed fabrics still exhibited substantial pHRR reductions compared with unmodified cotton. This demonstrates that, despite ten washing cycles, the fire-retardant effect of the combined silicon-phosphorus system remains significant and functionally preserved.

**Figure 11:**
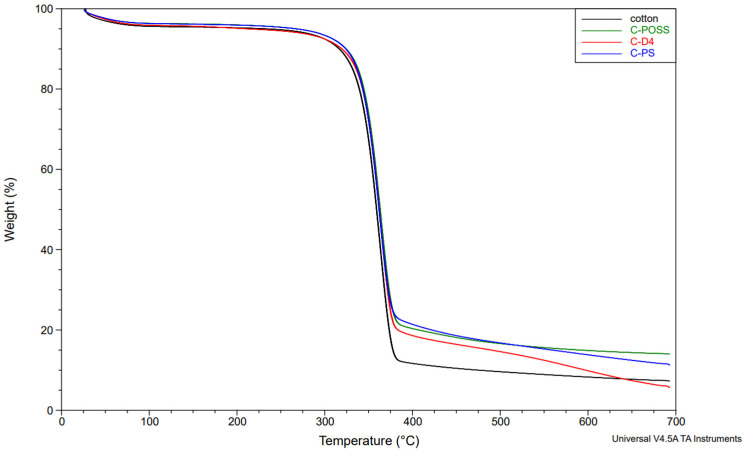
TG curves of samples in a nitrogen atmosphere before grafting phosphate onto the cotton surface.

**Figure 12:**
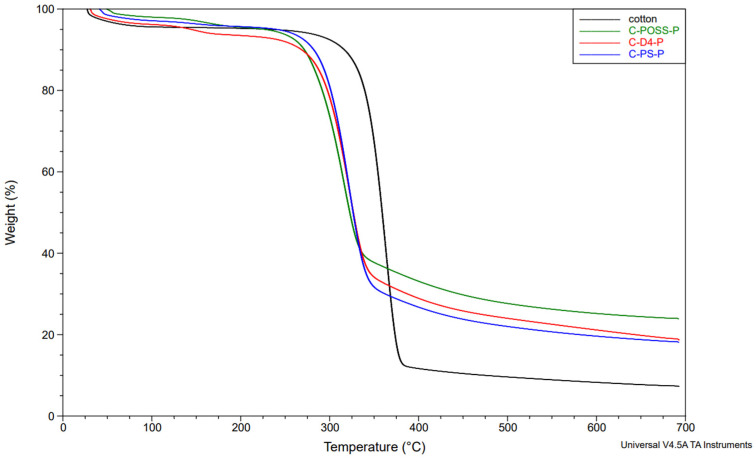
TG curves of samples in a nitrogen atmosphere after grafting phosphate onto the cotton surface.

**Figure 13:**
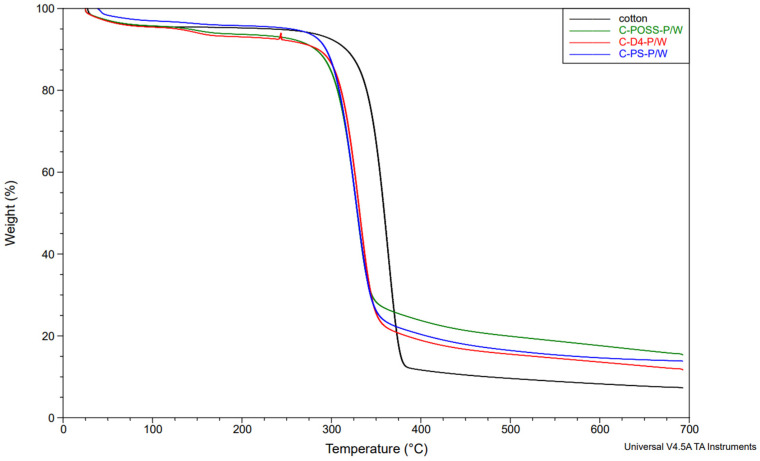
TG curves of samples in a nitrogen atmosphere after grafting phosphate onto the cotton surface and after the washing process.

In the next stage, the thermal stability of the modified fabrics was evaluated using thermogravimetric analysis (TGA) under a nitrogen atmosphere. The corresponding TGA results for the unwashed and washed samples are presented in Figure 11, Figure 12 and Figure 13.

**Figure 14:**
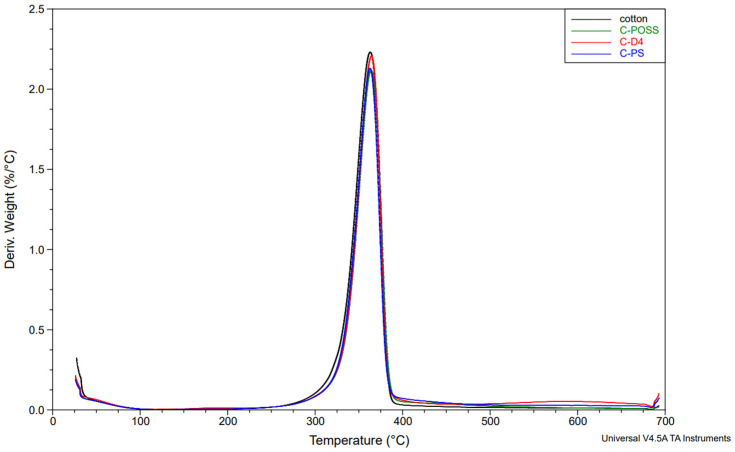
DTG profiles of samples under a nitrogen atmosphere before grafting phosphate onto the cotton surface.

**Figure 15:**
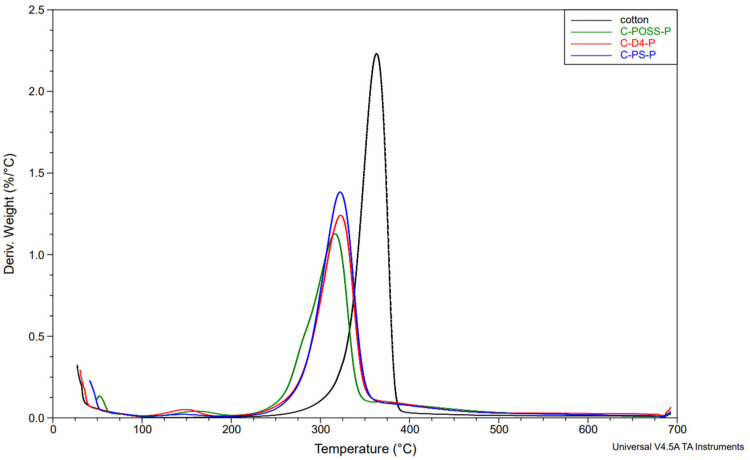
DTG profiles of samples under a nitrogen atmosphere after grafting phosphate onto the cotton surface.

**Figure 16:**
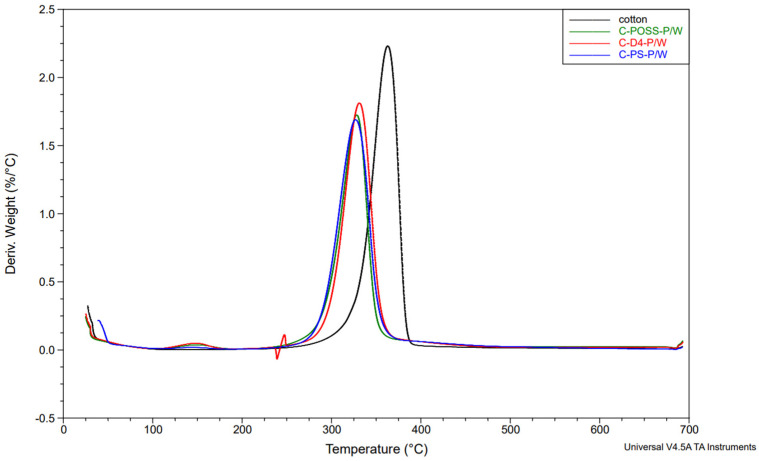
DTG profiles of samples under a nitrogen atmosphere after grafting phosphate onto the cotton surface and after the washing process.

Complementary information on the thermal degradation behavior was obtained from derivative thermogravimetry (DTG). The DTG curves for the unwashed and washed samples are shown in Figure 14, Figure 15 and Figure 16.

The thermal stability results of the modified samples before and after washing are presented in Table 4, which summarizes the key TGA and DTG parameters.

Neat cotton shows a single dominant degradation step with an onset temperature (Tₒₙₛₑₜ) of about 339 °C and a main DTG peak near 363 °C, leaving roughly 7–8 wt.% residue at 700 °C. For fabrics treated only with the organosilicon compounds (C-PS, C-D4, C-POSS), Tₒₙₛₑₜ and the temperature of the main DTG peak remain very close to those of the reference cotton (341–342 °C and 362–386 °C, respectively), indicating that the onset of thermal degradation is only slightly affected by the presence of the siloxane layer. However, these samples exhibit somewhat higher char yields, particularly C-POSS (14 wt.% residue) and C-PS (12 wt.%), which reflects the contribution of the inorganic Si-O-Si framework to the formation of non-combustible residue. The D4-modified fabric shows a smaller increase in residue (6 wt.%), consistent with its lower silicon content and more flexible structure compared with the POSS cage.

A different behavior is observed for the phosphorus-containing samples. Introduction of O,O′-diethyl dithiophosphate through the thiol-ene reaction (C-PS-P, C-D4-P, and C-POSS-P) leads to a marked decrease in Tₒₙₛₑₜ to about 284–295 °C and a shift in the main DTG peak to lower temperatures, while simultaneously increasing the amount of char to 18–24 wt.%. This earlier onset of mass loss is typical for phosphorus-based flame retardants and is associated with acid-catalyzed dehydration and crosslinking, which promote char formation at the expense of volatile flammable fragments. The highest residue is obtained for C-POSS-P (24 wt.%), again confirming the particularly effective combination of the highly inorganic POSS scaffold with the phosphorus/sulfur functionality, in line with literature data on P/Si systems where polysiloxanes or cage silsesquioxanes (POSS) enhance char stability.

After washing, the phosphorus-modified samples (C-PS-P/W, C-D4-P/W, and C-POSS-P/W) exhibit intermediate behavior: their onset and DTG peak temperatures shift back toward higher values, and the char residues decrease compared with the unwashed P-containing fabrics, but remain clearly above those of the samples modified only with organosilicon compounds. This pattern confirms partial leaching of phosphorus-containing species during laundering, while demonstrating that a significant fraction of the P/S modifier and the underlying siloxane network remains on the fibers and continues to provide enhanced thermal stability and char formation. The corresponding TGA curves for individual samples are provided in the Supplementary Information (Figures S1–S10).

**Figure 17:**
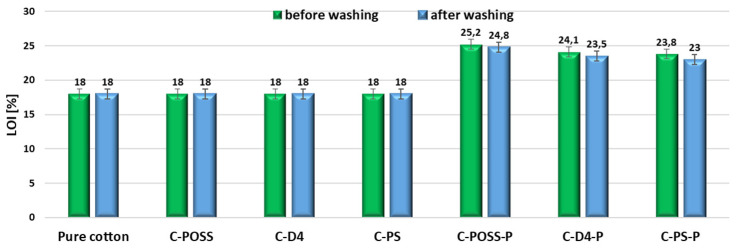
LOI results of modified samples before and after washing.

In the following step, the limiting oxygen index (LOI) of the modified fabrics was evaluated, both before and after washing, and the corresponding results are presented in Figure 17.

The limiting oxygen index (LOI) values obtained for the modified fabrics before and after washing are presented in Figure 11. Pure cotton exhibits an LOI of 18%, which is characteristic of cellulosic substrates and reflects their high flammability. Fabrics modified only with organosilicon compounds (C-POSS, C-D4, and C-PS) also show LOI values of 18%, comparable to the untreated fabric. This outcome is consistent with the expected behavior of silicon-based coatings. Although siloxane layers can act as thermal shields and promote char formation at elevated temperatures, they do not typically participate in gas-phase inhibition mechanisms. As a result, their presence alone does not markedly increase the oxygen concentration required to sustain combustion.

A significant improvement in flame resistance is observed for samples modified via the thiol-ene addition of O,O′-diethyl dithiophosphate. The LOI increases to 25.2% for C-POSS-P, 24.1% for C-D4-P, and 23.8% for C-PS-P, demonstrating the strong flame-retardant action of the phosphorus and sulfur groups incorporated onto the fiber surface. These values fall well within the range typically reported for phosphorus-containing finishes on cotton and indicate a substantial reduction in the minimum oxygen concentration required to sustain combustion. Among the three systems, the POSS-derived sample again exhibits the highest LOI, in agreement with the HRR and TGA results. The superior performance of the POSS-based modifier can be attributed to its cage structure, which enhances char formation and stabilizes the condensed-phase barrier generated during pyrolysis, thereby amplifying the effect of the phosphorus-containing layer. After washing, a slight decrease in LOI is observed for all P-modified samples, with values of 24.8% for C-POSS-P/W, 23.5% for C-D4-P/W, and 23.0% for C-PS-P/W. This moderate reduction is consistent with the partial leaching of O,O′-diethyl dithiophosphate shown by the EDS and TGA results. Nevertheless, the LOI values remain markedly higher than those of untreated cotton and the siloxane-only samples, confirming that the flame-retardant effect remains largely preserved even after ten laundering cycles.

SEM-EDS analysis revealed clear differences in surface composition before and after burning, confirming the formation of a protective residue during combustion (Table 2). Prior to burning, the modified fabric surface was dominated by carbon and oxygen from cellulose, with additional silicon, phosphorus, and sulfur originating from the organosilicon network and the dithiophosphate modifier. After burning, a pronounced increase in silicon and phosphorus content was observed, accompanied by a decrease in oxygen and sulfur. This trend indicates preferential retention and enrichment of thermally stable Si- and P-containing species within the residual char, while volatile degradation products are released during combustion. These changes support a flame-retardant mechanism dominated by condensed-phase action. Phosphorus-derived acidic species promote dehydration and carbonization of cellulose, whereas the siloxane framework undergoes further condensation and mineralization, leading to the formation of a silica- and phosphate-rich char layer. This inorganic-enriched residue acts as an effective barrier, limiting heat transfer, oxygen diffusion, and the release of flammable volatiles.

**Figure 18:**
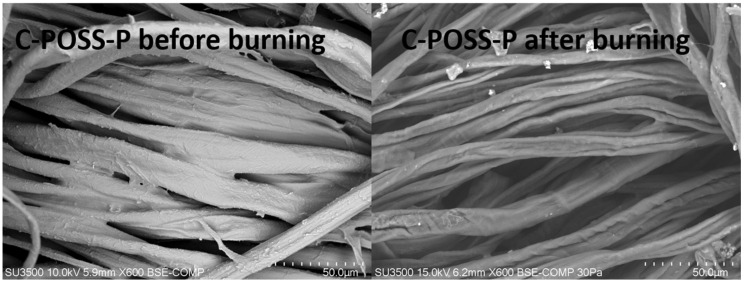
SEM image of sample C-POSS-P before burning (**left**) and after burning (**right**).

SEM images acquired after burning further corroborate this mechanism (Figure 18). The textile morphology remains recognizable, with individual fibers coated by a continuous, compact char layer. The absence of extensive cracking or fiber collapse indicates that the hybrid P/S-Si coating stabilizes the fabric structure during thermal exposure, consistent with the enhanced char yield and reduced heat release observed in TGA and MCC analyses.

**Figure 19:**
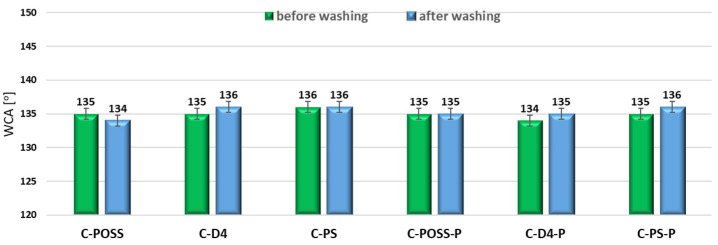
WCA of modified samples before and after washing.

Hydrophobicity of the modified fabrics was also evaluated by measuring the water contact angle (WCA) before and after washing, and the corresponding results are presented in Figure 19.

Figure 19 presents the water contact angle (WCA) values of the modified fabrics before and after washing. All treated samples exhibit WCA values in the range of 134–136°, demonstrating the successful introduction of hydrophobic siloxane-based layers onto the cotton surface. These values correspond to highly hydrophobic surfaces and are consistent with those reported previously for cotton modified with polysiloxanes, cyclic siloxanes, and silsesquioxanes (POSS) using thiol-ene or sol–gel-based chemistry. For samples modified exclusively with the organosilicon compounds (C-POSS, C-D4, and C-PS), the WCA values fall between 134 and 136°, indicating that all three modifiers effectively form low-energy surfaces. All three structures maintain sufficient surface coverage and methyl-rich orientation to achieve comparable hydrophobicity. The samples additionally modified with O,O′-diethyl dithiophosphate (C-POSS-P, C-D4-P, and C-PS-P) also remain highly hydrophobic. Their WCA values (134–136°) indicate that the grafting of the phosphorus-containing additive does not disrupt the siloxane-based hydrophobic layer.

After washing, the WCA values remain nearly unchanged, decreasing or increasing by only 1–2° depending on the sample. Such minor variations fall within experimental error and indicate excellent washing durability of the hydrophobic coating. This confirms the strong chemical anchoring of the alkoxysilyl-containing siloxane modifiers to cellulose and the stability of the crosslinked siloxane network. Even samples containing phosphorus—which showed partial leaching in EDS and TGA analyses—retain high hydrophobicity after washing.

**Figure 20:**
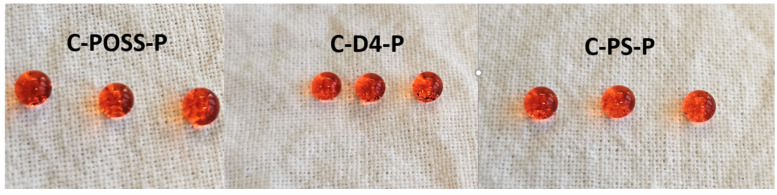
Images of water droplets at modified samples.

Photographs of water droplets placed on the modified fabric surfaces, illustrating the hydrophobic character of the obtained coatings, are presented in Figure 20.

## 4. Discussion

This study demonstrates that the structure of the organosilicon modifiers-vinyl derivatives of polysiloxane, cyclosiloxane, and silsesquioxane, in combination with thiol-ene grafting of O,O′-diethyl dithiophosphate, strongly influences the flame retardancy, hydrophobicity, and durability of cotton fabrics. The results confirm our working hypothesis that the inorganic character, molecular rigidity, and accessibility of vinyl groups govern both coating formation and post-functionalization efficiency. The coatings containing only organosilicon compounds showed similar add-on values and excellent washing durability, in agreement with our previous studies where alkoxysilane-bearing siloxanes formed stable Si-O-C linkages with cellulose. Although all three systems contained the same number of alkoxysilyl groups, elemental analysis revealed higher silicon content for the POSS-based coating, which results from the additional silicon atoms present in the functional groups attached at the corners of the Si_8_O_12_ cage rather than from the cage structure itself. Such structural differences translated into performance variations: POSS provided the densest siloxane network, D_4_ produced an intermediate rigidity, and linear polysiloxane formed the most flexible layer. These findings are consistent with earlier reports on silsesquioxane (POSS) and cyclic siloxane-modified textiles showing enhanced structural integrity due to increased inorganic fraction [39,40]. At the molecular level, the superior performance of the POSS-based system can be ascribed to the formation of a highly crosslinked, cage-derived siloxane network that yields a compact, silica-rich char with low permeability to heat and oxygen, while the globular geometry of the Si_8_O_12_ core maximizes the spatial separation and outward orientation of pendant vinyl/alkoxysilyl groups, facilitating both efficient condensation with cellulose and high thiol-ene grafting density of the phosphorus-containing thiol. By contrast, the cyclic D_4_ derivative generates a less densely crosslinked, vitrified Si-O-Si network, and the linear polysiloxane affords the most open, flexible architecture, which is more prone to softening and segmental motion during pyrolysis, resulting in a less cohesive char and a lower density of accessible vinyl sites for P/S grafting [41,42].

The incorporation of O,O′-diethyl dithiophosphate significantly improved flame retardancy. As expected from phosphorus-based systems and aligned with our earlier work. The modified fabrics exhibited reduced onset decomposition temperature, increased char yield, and strong pHRR suppression. The phosphorus/sulfur moiety promoted early dehydration and char formation, while silicon contributed to the stability of the resulting barrier. Among all systems, POSS-P showed the highest LOI, the lowest HRR, and the greatest char residue, demonstrating a strong P-Si synergy facilitated by the rigid POSS scaffold. D_4_-P and PS-P followed the same trend, consistent with their structural positioning between rigid and flexible organosilicon networks. The combined results of thermal analysis and microscale combustion calorimetry indicate that the flame-retardant action of the P/S-Si systems is governed predominantly by a condensed-phase mechanism. During heating, phosphoric and dithiophosphate species catalyze cellulose dehydration and promote crosslinking reactions. At the same time, the siloxane framework contributes to the formation of a thermally stable char enriched in silica and phosphate species. This char layer effectively shields the underlying fibers from heat and oxygen. The pronounced reduction in peak heat release rate, the shift in HRR maxima toward lower temperatures, and the substantial increase in residual, particularly for the C-POSS-P sample, are consistent with literature reports on P/S/Si-containing finishes applied to cotton and other polymeric substrates. In such systems, phosphorus-derived acidic species promote char formation, while silicon-based moieties reinforce and mineralize the residue. At the relatively low phosphorus loadings employed in this work, only a minor contribution from gas-phase radical trapping is expected [43,44,45].

The hierarchy C-POSS-P > C-D_4_-P > C-PS-P observed for LOI, pHRR reduction, and char yield indicates a strong structure-property relationship. The cage-like architecture of POSS enhances the spatial confinement of phosphorus-rich domains and promotes the formation of a continuous, ceramic-like surface layer. In contrast, the more flexible polysiloxane network allows partial cracking and fragmentation of the residue. This interpretation is consistent with observations reported for POSS- and polysiloxane-based flame-retardant coatings in other polymer systems. In addition, the higher local concentration and better accessibility of vinyl groups at the POSS periphery increase the efficiency of thiol-ene grafting. As a result, at comparable macroscopic add-on values, the POSS-based system can accommodate a higher density of phosphorus- and sulfur-containing functionalities per unit fabric area. This effect further strengthens condensed-phase charring and enhances pHRR suppression [41].

Hydrophobicity measurements revealed high and durable water contact angles in the range of 133–136° for all modified fabrics. This confirms that surface wettability is governed primarily by the siloxane layer rather than by the phosphorus-containing component. The minimal change in contact angle after laundering indicates strong covalent anchoring of the coating and is consistent with the durability observed in our earlier hydrophobization studies based on thiol-ene chemistry and sol–gel curing.

Overall, the results demonstrate that multifunctional performance can be achieved without compromising washing durability. The structural differences between the organosilicon precursors suggest a clear design strategy: POSS-based systems are most suitable for high-performance protective applications, D_4_-based systems for intermediate-level technical textiles, and linear polysiloxanes for cost-efficient surface treatments. The synergy between organosilicon networks and phosphorus-containing thiols highlights the potential of these coatings for protective clothing, upholstery, transport textiles, and composite reinforcement, where combined hydrophobicity and flame retardancy are required.

The washing-fastness results reveal a clear distinction between the behavior of the organosilicon scaffold and the phosphorus-containing fraction. While silicon content and add-on values remain essentially unchanged after ten laundering cycles, phosphorus and sulfur levels decrease measurably. This indicates that a fraction of the O,O′-diethyl dithiophosphate is physically trapped within the hybrid coating or weakly associated with the fiber surface and can therefore be removed by diffusion and leaching under aqueous, mildly alkaline washing conditions. Similar behavior has been reported for other phosphorus-based finishes on cotton, where non-reacted or loosely attached phosphate and phosphonate species are gradually lost during repeated laundering, leading to partial but not complete loss of flame retardancy [46,47]. In light of these observations, washing durability could be further improved by increasing the fraction of phosphorus species covalently integrated into the organosilicon network. This may be achieved by using co-monomers that enable a higher thiol-ene grafting density of O,O′-diethyl dithiophosphate and promote the formation of a more tightly crosslinked hybrid coating. Such an approach would limit the mobility and leaching of non-reacted phosphorus-containing fragments. Alternatively, future formulations could combine the present thiol-ene-anchored dithiophosphate with complementary reactive phosphorus derivatives capable of forming additional stable linkages, such as P-N-C bonds, to cellulose or to other components of the finish, as reported for durable phosphoramidate- and ammonium phosphoric acid-based systems [48,49]. Future research may also focus on increasing phosphorus grafting efficiency by reducing steric hindrance around vinyl groups, exploring bio-derived flame retardants compatible with thiol-ene chemistry, and extending this approach to other fibers such as flax, hemp, viscose, or polyester. Additional studies on UV aging, mechanical durability, and the integration of nitrogen-containing modifiers could further enhance multifunctionality and broaden the scope of practical applications.

## 5. Conclusions

This study demonstrates that cotton fabrics can be effectively functionalized to achieve simultaneous hydrophobicity and flame retardancy through a two-step process involving deposition of alkoxysilyl-functional organosilicon compounds and subsequent thiol-ene grafting of O,O′-diethyl dithiophosphate. All three organosilicon systems-polysiloxane, cyclotetrasiloxane (D_4_-Vi), and silsesquioxane (POSS)-formed uniform and durable coatings, with add-on values of 8.6–9.2% that remained nearly unchanged after washing. The modified fabrics exhibited high hydrophobicity, with water contact angles of 134–136°, which were preserved after ten laundering cycles, confirming the robustness of the siloxane networks covalently anchored to cellulose. Flame retardancy improved markedly after thiol-ene grafting of the phosphorus-containing compound. The peak heat release rate decreased from 319 W/g for raw cotton to 131 W/g for C-PS-P, 121 W/g for C-D4-P, and 108 W/g for C-POSS-P, demonstrating up to a 66% reduction. The limiting oxygen index increased from 18% (untreated cotton) to 23.8–25.2% for phosphorus-modified samples, confirming significantly enhanced resistance to sustained combustion. Thermogravimetric analysis further showed increased char residue, from 7 to 8 wt.% for untreated cotton to 18 wt.% for C-PS-P, 20 wt.% for C-D4-P, and 24 wt.% for C-POSS-P-along with shifts in decomposition profiles characteristic of phosphorus-induced condensed-phase stabilization. Although washing caused partial leaching of the phosphorus modifier, the washed samples maintained substantially reduced pHRR values and elevated LOI and char yields compared with untreated cotton, demonstrating long-lasting flame-retardant performance.

Among all systems, the POSS-based modifier provided the greatest overall improvement, which is attributed to its higher inorganic content and favorable spatial arrangement of reactive groups that enhance both siloxane network formation and phosphorus grafting efficiency. The D_4_-based system showed intermediate performance, while the polysiloxane-based coating resulted in the lowest, but still significant, enhancement relative to the unmodified fabric. Overall, the results show that the developed fluorine-free modification strategy enables the creation of cotton textiles with durable hydrophobicity and substantially improved flame retardancy. The approach is versatile, tunable through the choice of organosilicon architecture, and transferable to other natural and synthetic fibers, offering potential applications in protective clothing, transport textiles, interior furnishings, and multifunctional composite materials. Overall, the results confirm that multifunctional performance can be achieved without compromising wash durability. The structural differences between the organosilicon precursors provide a clear design strategy: POSS for high-performance protective applications, D_4_ for intermediate-level technical textiles, and polysiloxane for cost-efficient treatments.

## Figures and Tables

**Table 1:** Add-on value of modified samples before and after washing.

Symbol	Modification	Add-on Value Before Washing [%]	Add-on Value After Washing [%]
C-PS	Polysiloxane	8.8	8.8
C-POSS	Silsesquioxane	9.2	9.2
C-D4	Cyclosiloxane	8.7	8.6
C-PS-P	Polysiloxane + phosphorene	13.9	12.5
C-POSS-P	Silsesquioxane + phosphorene	15.3	13.7
C-D4-P	Cyclosiloxane + phosphorene	13.7	12.4

**Table 2:** SEM-EDS results of modified samples before and after washing.

Sample	Elemental Composition Before Washing					Elemental Composition After Washing				
	C (at %)	O (at %)	Si (at %)	P (at %)	S (at %)	C (at %)	O (at %)	Si (at %)	P (at %)	S (at %)
Cotton	0.0	0.0	-	-	-	0.0	0.0	-	-	-
C-PS	33.5	59.7	4.6	-	2.0	33.5	59.7	4.5	-	2.0
C-D4	33.3	60.5	4.1	-	2.0	33.3	60.5	4.1	-	2.1
C-POSS	31.9	59.9	6.4	-	1.6	31.9	59.9	6.4	-	1.6
C-PS-P	33.4	57.9	4.2	1.1	3.4	33.3	58.4	4.3	0.7	3.0
C-D4-P	34.7	57.6	3.9	0.9	3.0	34.6	57.8	4	0.7	2.7
C-POSS-P	32.3	55.5	5.6	1.9	4.8	31.3	57.7	5.7	1.2	3.9
C-POSS-P after burning	42.5	34.6	14.6	5.5	2.8	-	-	-	-	-

**Table 3:** MCC results before and after washing.

Sample	MCC Results Before Washing			MCC Results After Washing		
	pHRR [W/g]	Tmax [℃]	t at pHRR [s]	pHRR [W/g]	Tmax [℃]	t at pHRR [s]
Cotton	304	376	354	-	-	-
C-PS	298	373	348	-	-	-
C-D4	292	372	348	-	-	-
C-POSS	288	369	347	-	-	-
C-PS-P	132	300	280	189	315	286
C-D4-P	122	300	272	186	317	282
C-POSS-P	108	296	261	185	317	277

**Table 4:** Thermal stability results of modified samples.

Sample	T_onset_ [°C]	DTG_temp_ [°C]	DTG_peak_	Stage I	Stage II		Stage III		Residue at 700 °C [°C}
				Mass Loss [%]	Start Temp [°C]	Mass Loss [%]	Start Temp [°C]	Mass Loss [%]	
Cotton	338.84	363.04	2.231	11.85	325.67	73.99	380.67	5.849	7.378
C-POSS	341.46	364.45	2.113	10.76	326.38	66.11	379.26	9.11	14.02
C-POSS-P	283.54	315.8	1.128	6.282	248.82	55.76	344.01	14.55	24.02
C-POSS-P/W	304.9	328.49	1.724	8.421	276.32	64.07	359.52	11.38	15.42
C-D4	341.72	363.04	2.199	10.26	320.74	70	385.61	13.75	5.96
C-D4-P	293.95	323.56	1.239	8.705	257.28	57.65	352.47	14.71	19.01
C-D4-P/W	313.56	330.71	1.813	9.67	282.83	67.85	359.29	10.66	11.88
C-PS	340.62	361.63	2.126	9.66	322.85	67.24	382.79	11.37	11.59
C-PS-P	294.29	322.15	1.384	12.29	288.3	56.89	355.29	12.7	18.13
C-PS-P/W	303.16	327.08	1.69	10.67	295.36	66.13	363.04	9.302	13.89

## Data Availability

The original contributions presented in this study are included in the article/Supplementary Materials. Further inquiries can be directed to the corresponding authors.

## Supplementary Materials

The following supporting information can be downloaded at: https://www.mdpi.com/article/10.3390/ma19020265/s1, Figure S1: TGA of pure cotton; Figure S2: TGA of sample C-POSS; Figure S3: TGA of sample C-POSS-P; Figure S4: TGA of sample C-POSS-P/W; Figure S5: TGA of sample C-D4; Figure S6: TGA of sample C-D4-P; Figure S7: TGA of sample C-D4-P/W; Figure S8: TGA of sample C-PS; Figure S9: TGA of sample C-PS-P; Figure S10: TGA of sample C-PS-P/W.
