# Supplementary material for: Flame-Retardant and Hydrophobic Cotton via Alkoxysilyl-Functionalized Polysiloxanes, Cyclosiloxanes, and POSS with Surface Thiol-Ene Dithiophosphate Grafting
Source: Materials (Basel). 2026 Jan 8;19(2):265. doi: 10.3390/ma19020265 (PMC12842728; doi:10.3390/ma19020265)
Supplement: Supplementary file 1 [file materials-19-00265-s001.zip › materials-4046721-supplementary.pdf]

materials

Supplementary Materials

Flame-Retardant and Hydrophobic Cotton via Alkoxysilyl-Functionalized Polysiloxanes, Cyclosiloxanes and POSS with Surface Thiol-Ene Dithiophosphate Grafting

TGA vs DTG

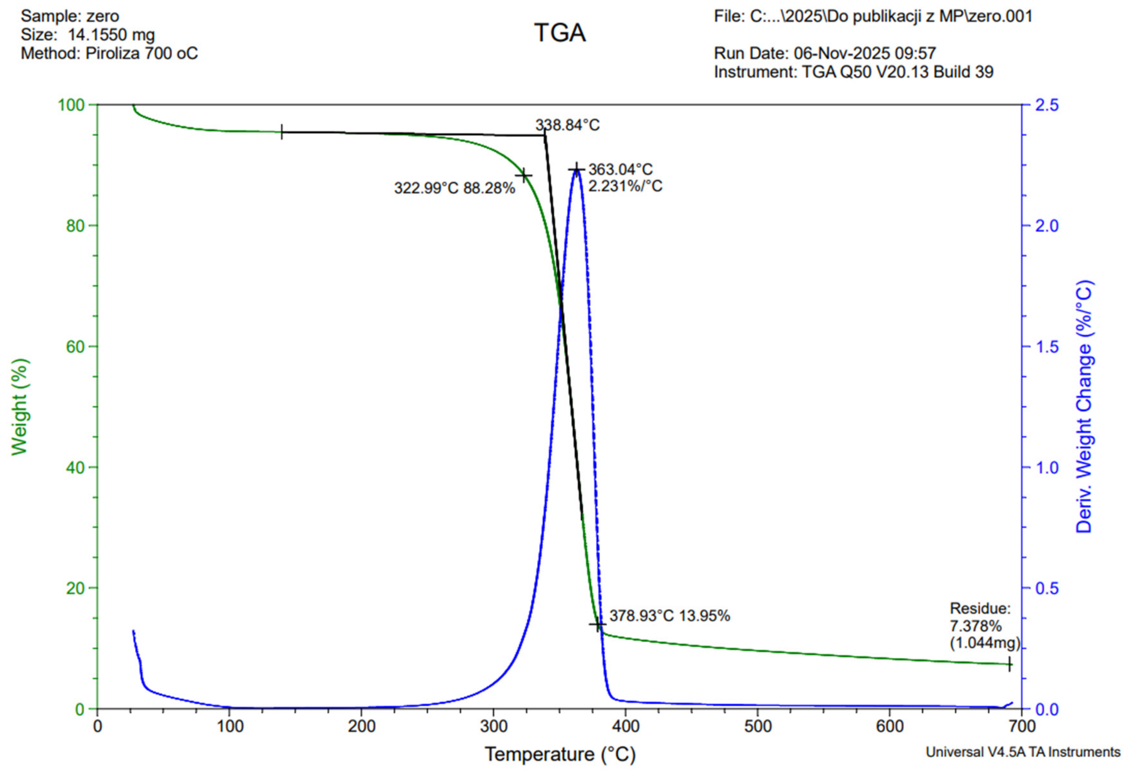

Figure S1. TGA of pure cotton.

 https://doi.org/10.3390/ma19020265

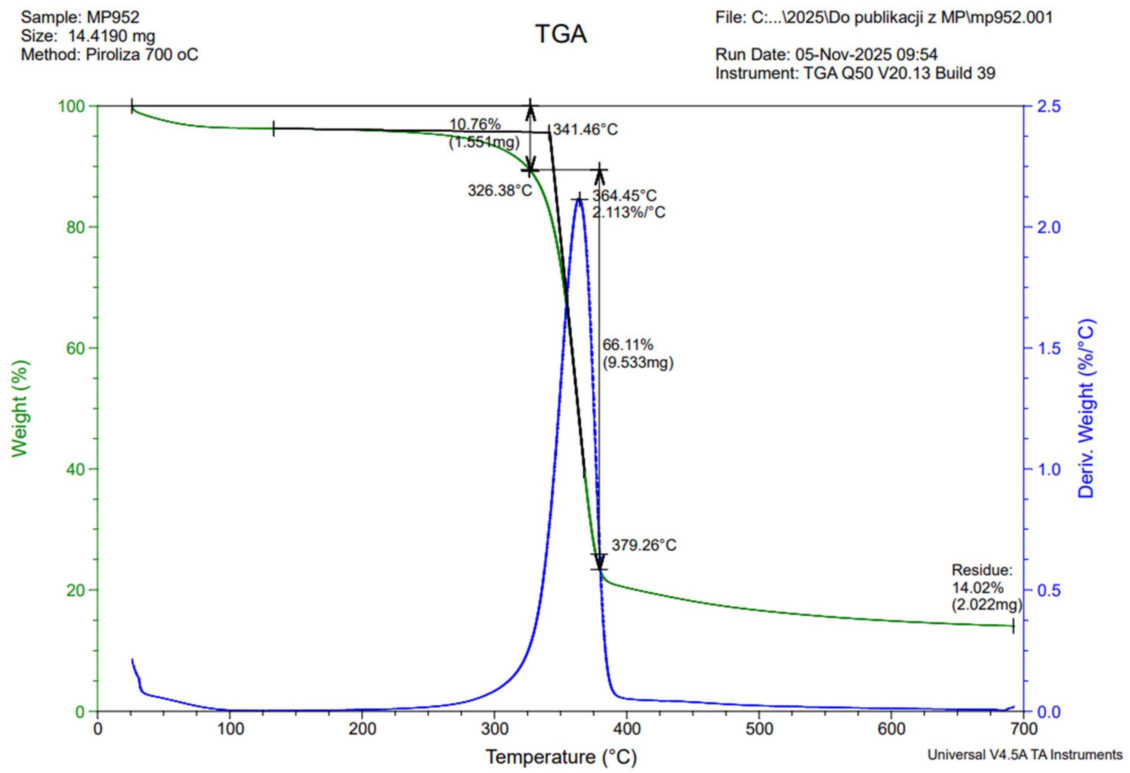

Figure S2. TGA of sample C-POSS.

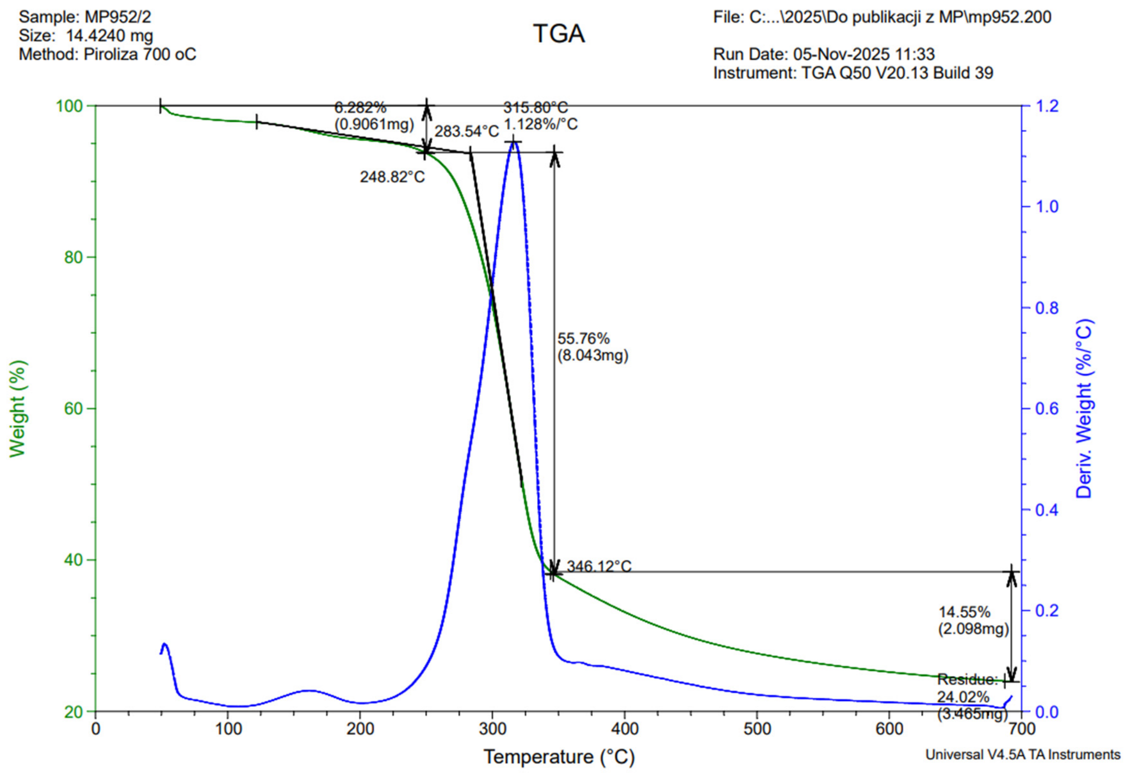

Figure S3. TGA of sample C-POSS-P.

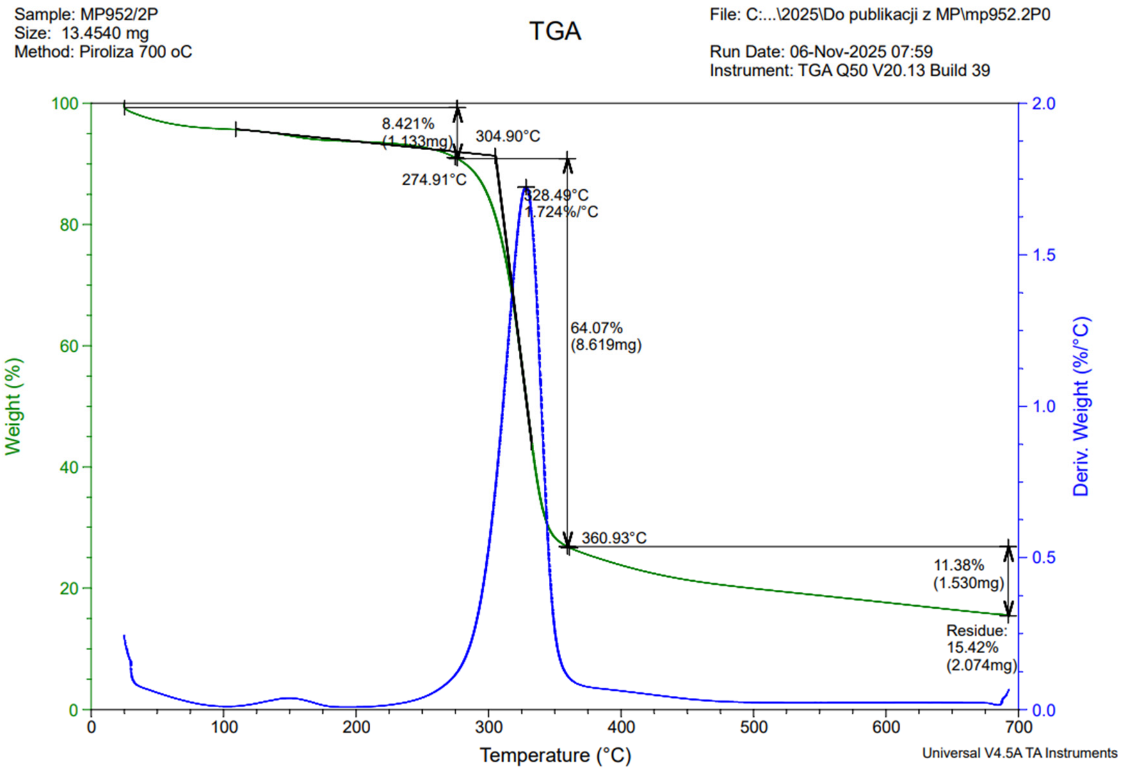

Figure S4. TGA of sample C-POSS-P/W.

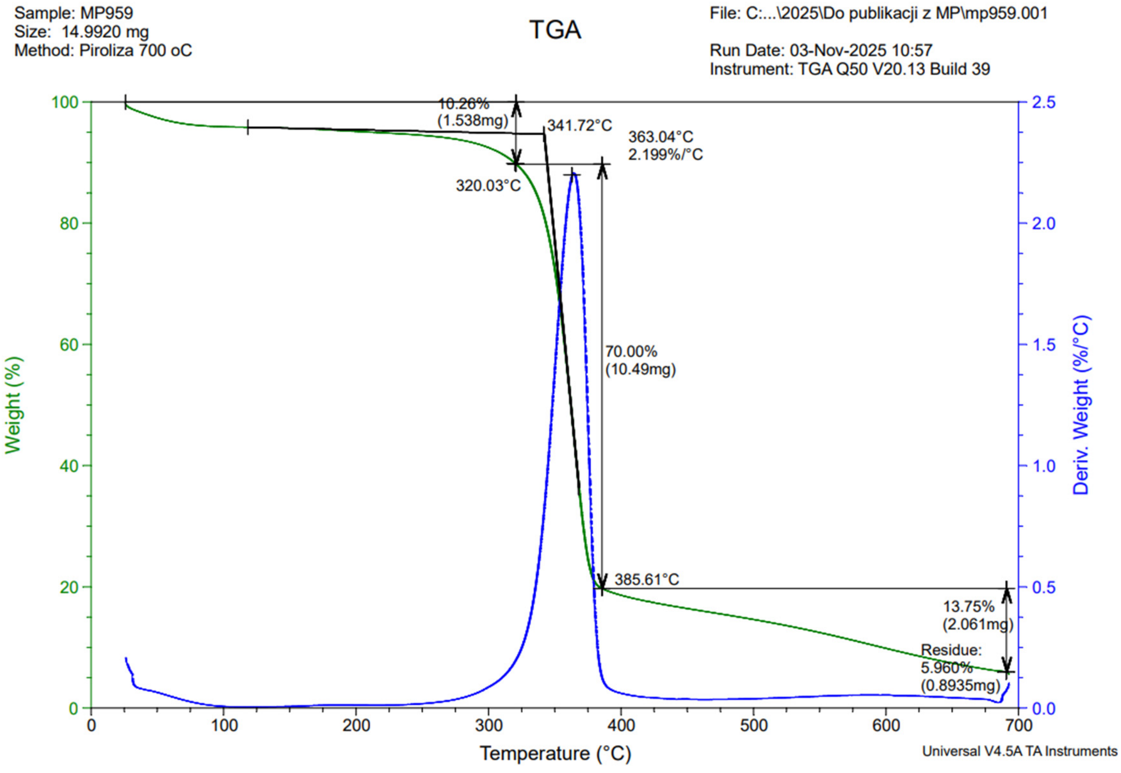

Figure S5. TGA of sample C-D4.

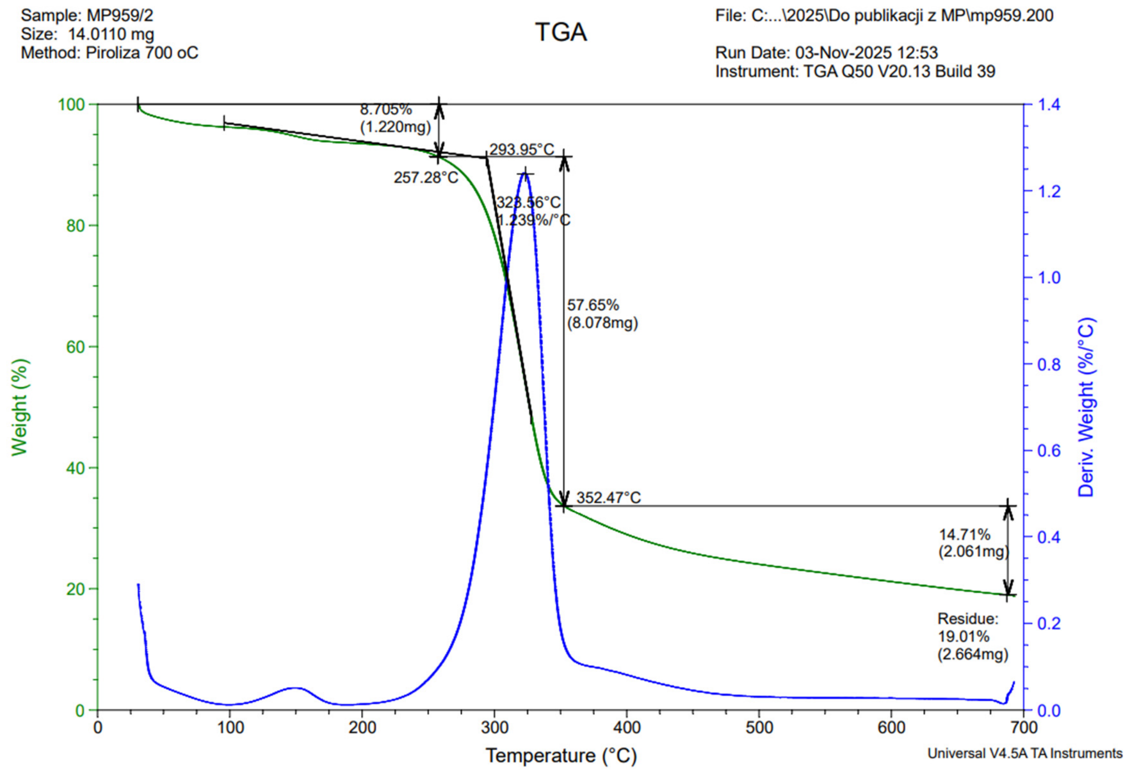

Figure S6. TGA of sample C-D4-P.

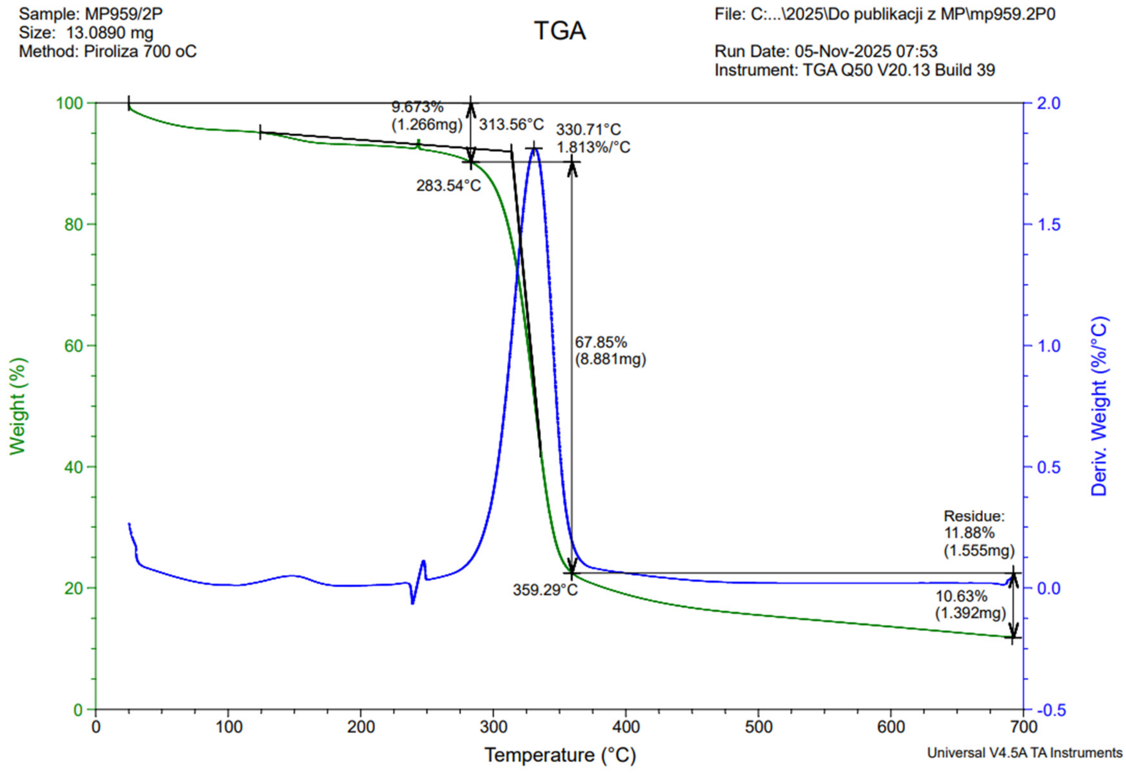

Figure S7. TGA of sample C-D4-P/W.

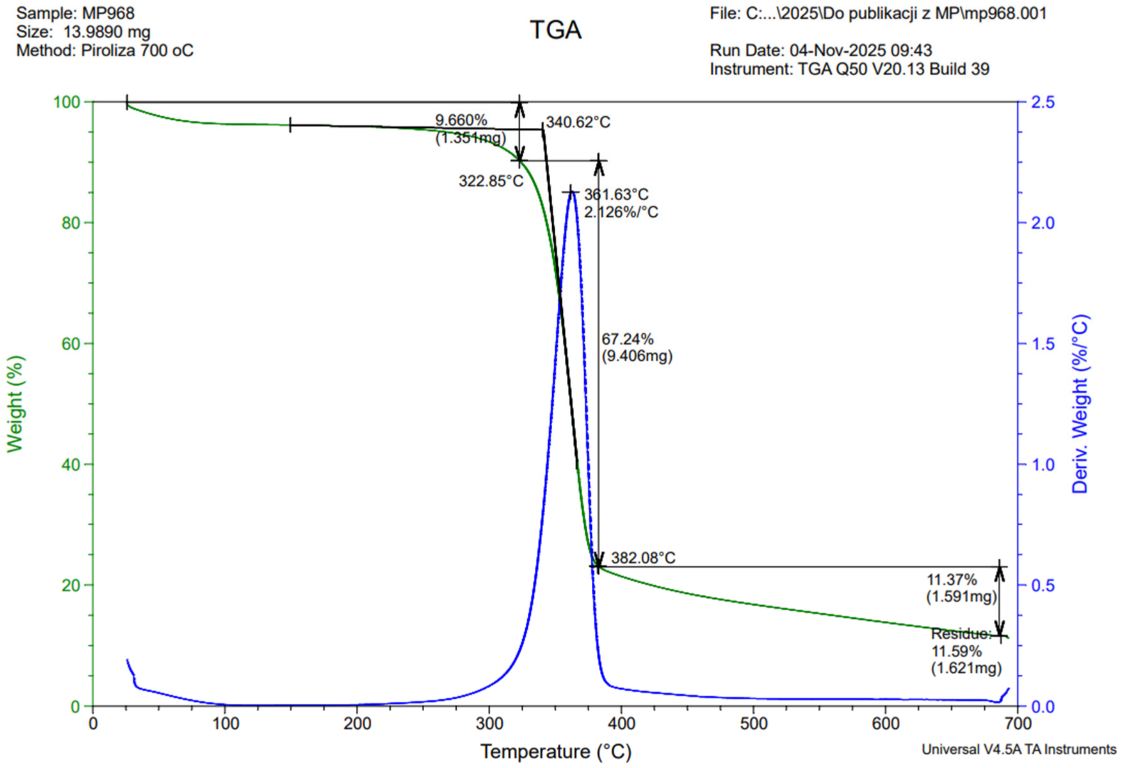

Figure S8. TGA of sample C-PS.

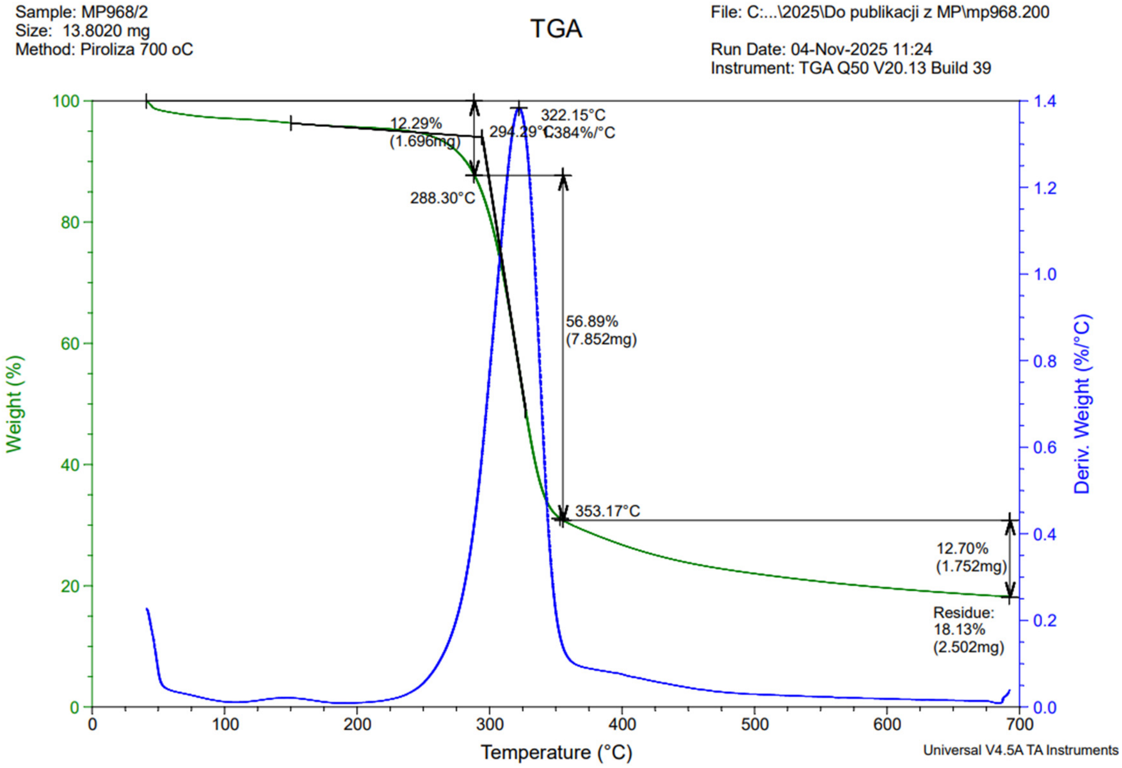

Figure S9. TGA of sample C-PS-P.

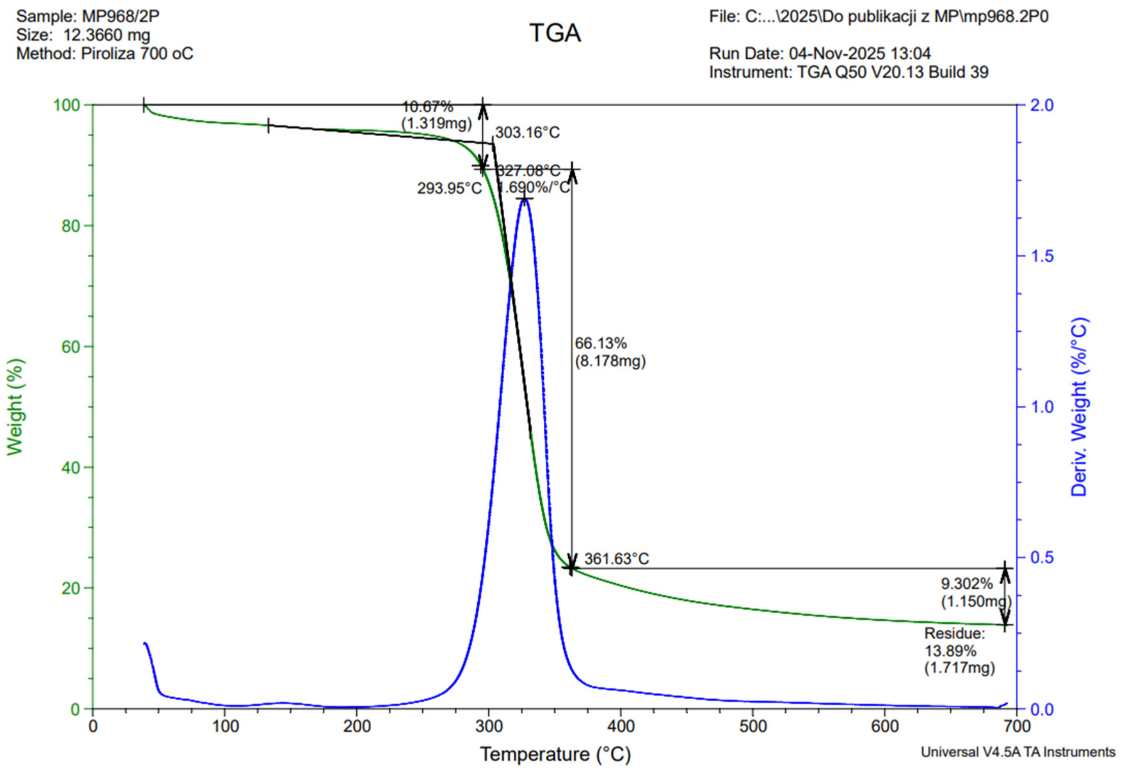

Figure S10. TGA of sample C-PS-P/W.
